# Varicocele: An Endocrinological Perspective

**DOI:** 10.3389/frph.2022.863695

**Published:** 2022-04-01

**Authors:** Giuseppe Bellastella, Raffaela Carotenuto, Francesco Caiazzo, Miriam Longo, Paolo Cirillo, Lorenzo Scappaticcio, Carla Carbone, Davide Arcaniolo, Maria Ida Maiorino, Katherine Esposito

**Affiliations:** ^1^Unit of Endocrinology and Metabolic Diseases, Department of Advanced Medical and Surgical Sciences, University of Campania “Luigi Vanvitelli,” Naples, Italy; ^2^Urology Unit, Department of Woman Child and of General and Specialist Surgery, University of Campania “Luigi Vanvitelli,” Naples, Italy

**Keywords:** varicocele, hormones, androgen, testosterone, gonadotropins

## Abstract

Varicocele affects 15% of male population but it is more frequently identified in patients searching medical care for infertility. The impact of varicocele on semen production and fertility is known, but the relationship between clinical varicocele and impaired hormonal production is not clear. In published literature there are some studies regarding hormonal alterations in patients with varicocele but no review in which all the hormonal findings are explained. The aim of this review is to evaluate, by most common search engine, what is known about hormonal alterations in varicocele-bearing patients, to verify if a cause-effect relationship is documented and to give a useful contribution to in clinical management of this kind of patients. We found contradictory results about hormonal status from literature. Some studies confirmed a decrease of testosterone levels and higher FSH and LH levels that normalize after varicocelectomy, others found lower than normal levels of dihydrotestosterone due to decreased activity of epididymal 5-α-reductase. Lower circulating Anti-Müllerian Hormone levels, accompanied by a decreased Inhibin-B level, were reported as indicators of the decreased Sertoli cells function in varicocele-bearing adult patients. The finding of higher basal 17-OH-progesterone concentrations in patients with varicocele was explained by some authors with a testicular C-17,20-lyase deficiency. There is no doubt that varicocele could led to hormonal alterations. This review proposes that the impaired free sexual steroid levels are the result of a slight, deep-rooted defect in the testes of a certain amount of men with varicocele but further multicentre, randomized controlled studies remain mandatory to better clarify the hormonal features of patients with varicocele and to assess the utility of hormonal evaluation for establishing the duration of varicocele and for better identifying patients who need surgical correction.

## Introduction

Varicocele is an irregular dilation of the pampiniform plexus determining reflux of venous blood flow. It affects around 15% of male population but it is more frequently identified in patients searching medical care for infertility (1, 2). It has been considered for a long time as one of the most frequent diseases affecting fertility potential, and the most common surgically curable cause of male sterility. However, a recent multicenter worldwide study encouraged by the European Academy of Andrology (3, 4) reported in men without any health or fertility problems a high incidence of varicocele (~37%) similar to men with primary infertility (5–7). These records propose that it could have lower effect on semen alteration, and its treatment have to be limited to selected populations. In accordance, ongoing EAU Guidelines on Male Infertility support specific indications for varicocele surgical correction both in adults and adolescents. In particular treating infertile men with clinical varicocele, abnormal semen parameters, or otherwise unexplained infertility in a couple in which the female partner has a good ovarian reserve, to improve the fertility rate, is considered a strong recommendation. On the contrary, there is a weak recommendation to treat men with raised sperm DNA fragmentation or who have suffered from failed assisted reproductive techniques (8).

Ultrasound (US) is the instrumental test of choice for diagnosing varicocele (8–10). Varicoceles are initially identified during physical examination. Color Doppler ultrasound can confirm the diagnosis and provide additional information on the presence of venous reflux and venous diameter (8–10).

There is a correlation among male sterility, ipsilateral testicular atrophy, and clinically detectable, rather than non-detectable, varicocele (11). In agreement with the criteria introduced in 1970 by Dubin and Amelar, varicocele is diagnosed and scored clinically in three grades (12). Grade 1 varicocele is palpable only while standing during Valsalva maneuver; Grade 2 is palpable also at rest while standing; Grade 3 is visible through the skin of scrotus. Among men evaluated for infertility varicocele is still the most frequent finding, identified in 35% of men with primary sterility and 70–80% of men with secondary sterility (13).

The impact of varicocele on semen production and fertility is known, but the relationship between clinical varicocele and impaired hormonal production is not clear; it has been hypothesized that the degree of varicocele may be inversely correlated with testosterone production (13, 14). The aim of the present review was to elucidate the hormonal features of patients with varicocele.

## Methods

A systematic Pubmed engine search was carried by using a set of keywords as follows: “varicocele” and “hormone,” “testosterone,” “gonadotropins,” “estradiol,” “androgens;” original papers and metanalyses reporting results on hormonal impairment in terms of testosterone, gonadotrophins and other hormones in patients with varicocele were selected and the contents critically evaluated.

### Pathophysiological Aspects of Varicocele and Gonadal Function

In recent years, many studies have clarified the etiological and pathophysiological aspects of varicocele (15) and its impact on spermatogenesis and hormonal axis. Various hypotheses have been proposed to describe the negative effect of varicocele on testicular function. The suggested etiology of varicocele-induced effects includes anatomical changes such as venous pooling, higher scrotal temperature, oxidative stress, hypoxia, backflow of metabolites from the adrenal gland, increase of CO_2_ and nitric oxide, autoimmunity, and damage of Leydig cells (16–18).

Testicular function is temperature-dependent; venous stasis causes an increase in scrotal temperature, a reduction of Sertoli cell function, an abnormal testicular protein metabolism, and a reduction in Leydig cell testosterone production (9), due to reduction of precursor conversion (19).

**Figure 1:**
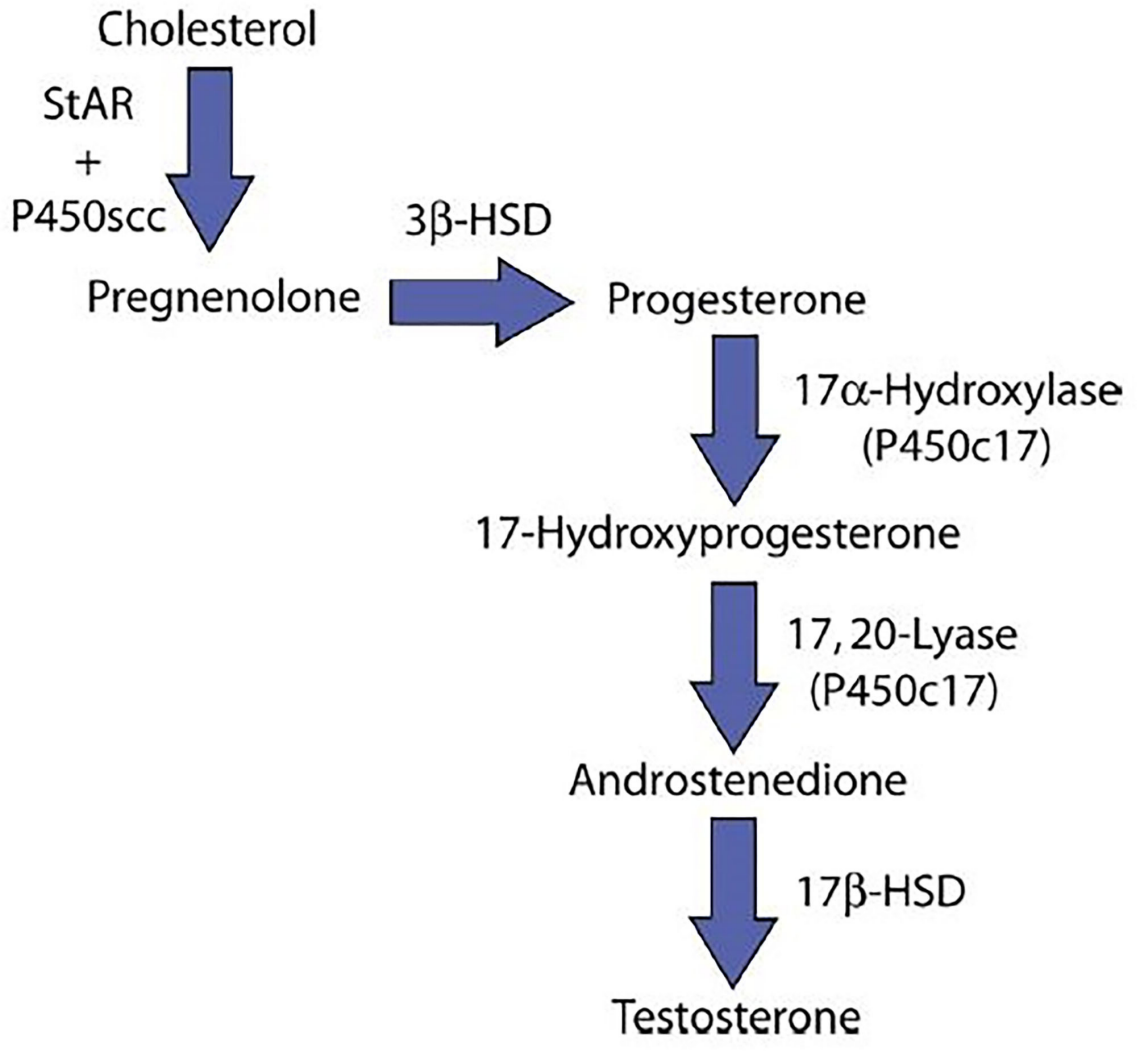
Testosterone synthesis. StAR, steroidogenic acute regulatory protein; P450scc, Cholesterol side-chain cleavage enzyme; 3β-HSD, 3β-Hydroxysteroid dehydrogenase; 17β-HSD, 17β-Hydroxysteroid dehydrogenase; P450c17, Cytochrome P450 17A1.

Results from a study with a sensitive assay system that calculated the activities of the five enzymes in the testosterone biosynthetic pathway showed that testosterone production was inhibited at the 17-α-hydroxylase step (20) (Figure 1). On the other hand, some studies have demonstrated an inhibition of testicular C-17,20-lyase activity, enzyme involved in testosterone production (21) (Figure 1). In Leydig cells, Luteinizing Hormone (LH) stimulates steroidogenesis by triggering the cascade of events at mitochondrial level leading from cholesterol to testosterone. Follicle Stimulating Hormone (FSH) is the main growth stimulator of seminiferous tubules starting from puberty. Varicocele increase internal scrotal temperature and can cause decrease of testosterone synthesis by Leydig cells, reduction of Sertoli cells function and germinal cells damage. Sertoli cells are also the principal site of production of Inhibin-B (Inh-B) that acts as a circulating feedback modulator of FSH secretion by the pituitary gland. Many studies demonstrated an inverse correlation between circulating Inh-B and FSH in fertile and infertile men; this would explain the rise of FSH levels in men with varicocele. Inh-B secretion is determined by the interaction between Sertoli cells and spermatids; in fact they influence Inh-B production and are sensitive to hyperthermia (22). The rise of scrotal temperature caused by varicocele impairs spermatids function, reducing Inh-B production (23).

**Figure 2:**
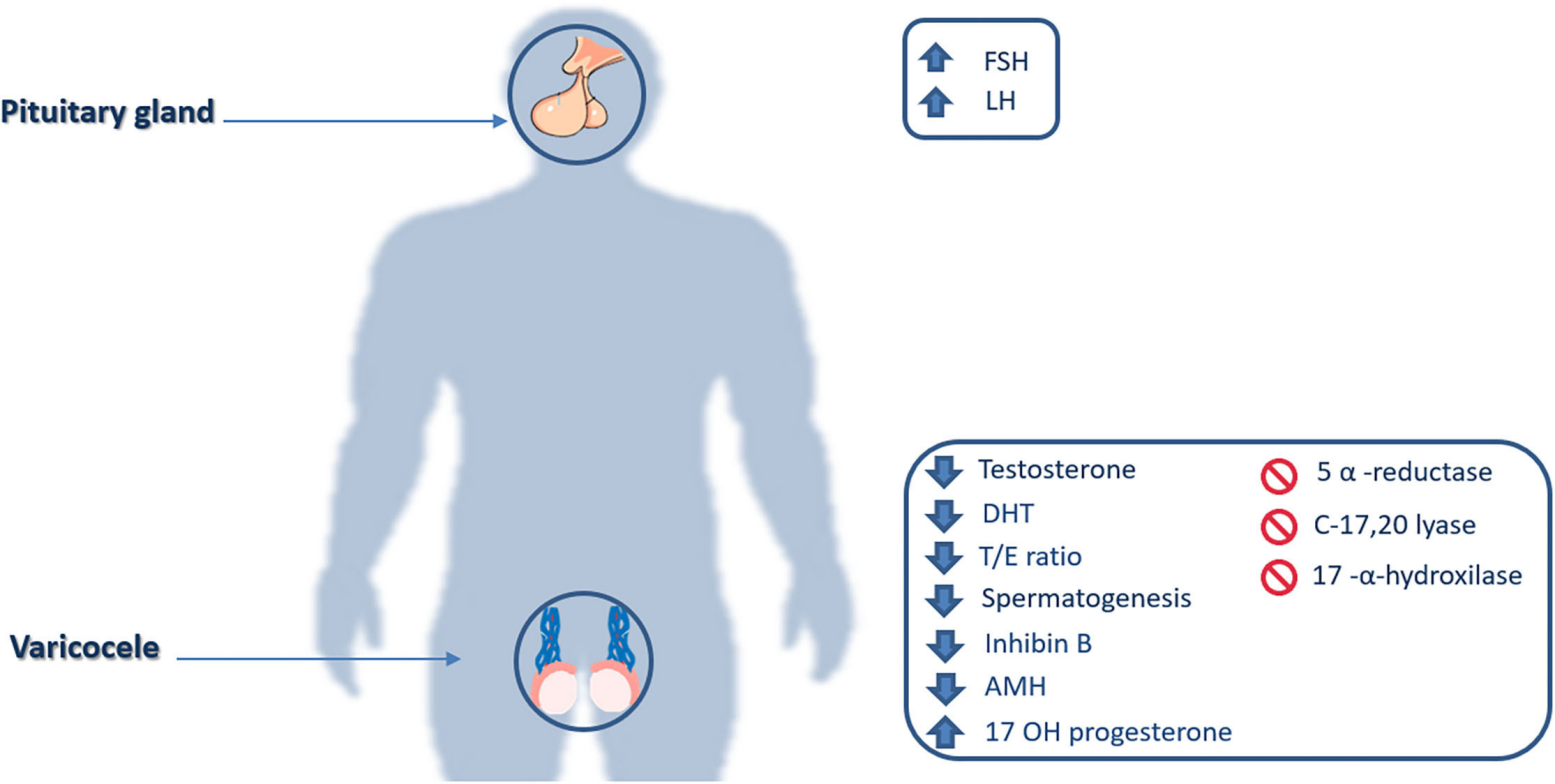
Hormonal and enzymatic impairments. FSH, Follicle-stimulating hormone; LH, Luteinizing hormone; DHT, Dihydrotestosterone; T/E ratio, Testosterone/estradiol ratio; AMH, Anti-Müllerian hormone; 17 OH Progesterone, 17α-Hydroxyprogesterone.

Leydig cells are the testicular site of testosterone production and their possible damage by varicocele toxic environment, may lead to a decrease of testosterone and rise of LH serum levels, due to the lack of negative feedback to pituitary gonadotrophic cells (Figure 2).

### Testosterone and Varicocele

A certain amount of evidence suggests that varicocele impairs testicular Leydig cells function with, in addition to its probable effects on semen alteration, a meaningful decrease in testosterone production (24, 25). Although the consequences of varicocele and its resolution have been described for decades, only few studies have been conducted in this field, often retrospective, and therefore with limited value even because with contradictory results (26–28).

A meta-analysis by Li et al. which included most of these studies, evaluated the effect of surgical varicocele repair on improving Leydig cells function. This was measured by the change in testosterone concentration after varicocelectomy. On the basis of nine selected studies, including 814 patients, the combined analysis indicated that mean serum testosterone levels after surgical treatment increased by 97.5 ng/dL (95% CI 43.7– 51, *P* = 0.0004) compared with preoperative levels. The authors concluded that varicocele causes a disturbance in Leydig cells function resulting in decreased testosterone biosynthesis, and that surgical repair significantly increases testosterone levels in men with varicocele (26).

On the other side, results from other trials that evaluated the hormonal axis in varicocele-bearing patients did not show a significant decrease in basal testosterone but only a significant decrease in dihydrotestosterone (DHT) concentrations, likely caused by diminished activity of epididymal 5-alpha-reductase in patients with varicocele (29, 30).

Different studies demonstrated the relationship between varicocele repair and testosterone levels, suggesting that varicocelectomy leads to an enhancement of testosterone production up to eugonadal levels, significant for men of all age groups (31, 32). A recent study including 100 patients (50 hypogonadal and 50 eugonadal) with varicocele of all grades, showed a significant difference in pre- and post-varicocelectomy testosterone concentrations in hypogonadal patients but not in eugonadal ones (33).

### LH, FSH, and Varicocele

Many studies demonstrated changes of serum FSH and LH in men with varicocele, but the reasons are not clear. Nine clinical trials evaluated alteration of serum FSH and LH levels in a cohort of 312 patients before and after varicocelectomy. The results suggested that FSH levels were more elevated pre-operatively than post-operatively (*P* < 0.001). The average reduction of serum FSH was from 0.1 to 4.8 ng/dL. On the other hand, also LH levels significantly decreased after surgery with respect to the higher pre-operatory levels (*P* < 0.0005). The average decrease of the serum LH was from 0.2 to 2.1 ng/dL (34). A study including fifty-five patients with varicocele and infertility showed that 16% of patients had basal FSH levels above the normal confidence limits with a significant difference between the control and the varicocele group. On the other hand, just 8% of the people exhibited high basal LH levels; however, in the severely oligozoospermic patients (*n* = 11), LH was significantly higher (*Z* = 2.190; *p* < 0.05) than in normozoospermic men (35). Moreover, a statistically significant difference between pre- and post-varicocelectomy LH concentration was found in hypogonadal patients more than in eugonadal ones, whereas no significant difference in FSH concentrations was found (33). In a case-control study, Garolla et al. evaluated 40 patients with left varicocele, 20 obese men and 20 healthy control subjects for a series of parameters such as testicular volume, hormones, sperm parameter and 24-h scrotal temperature monitoring by a cutaneous thermochip.

The results showed a statistically significant rise in 24-h mean scrotal temperature in obese men and men with varicocele compared with controls (P < 0.01 for both). Higher scrotal temperature was associated with impaired sperm quality and with higher FSH plasma levels. In fact, although FSH, LH and testosterone levels in varicocele and obese men were in the normal range, FSH concentration was significantly higher in these two groups than in controls (P < 0.001) (36).

Recently, a finding of strong correlation between FSH concentrations and varicocele in a pediatric population was reported. High FSH levels and low testicular volume in young patients could be a marker of varicocelectomy timing, expecially if semen analysis could not be assessed (37, 38).

### Other Hormones and Varicocele

Anti-Müllerian Hormone (AMH) and Inh-B were evaluated in a study considering subfertile adult men with varicocele. Circulating AMH levels were 60% lower in subfertile subjects than in controls, accompanied by reduced level of Inh-B, caused by a damage of Sertoli cells in men with varicocele (39). On the contrary, in pre-pubertal and pubertal boys with varicocele, AMH and Inh-B levels were higher, denoting a compensatory increase in Sertoli cells function in the early-onset of varicocele (40). Another study compared the AMH concentrations in the local spermatic vein and in peripheral blood. Concentration of AMH in the local spermatic vein was lower than in the peripheral blood, indicating that a poor blood supply in varicocele-bearing patient causes the degeneration of Sertoli cells function (41).

Scholler et al. found a significantly more elevated basal concentrations of 17-OH-progesterone in varicocele-bearing patients, confirming the suggested deficiency of testicular C-17,20-lyase, due to the severe dependence of this cytochrome P450-XVII-dependent enzyme on the scrotal temperature (42, 43).

The effect of varicocele on serum testosterone: estradiol (T:E) ratio and the result of varicocelectomy was also explored. In varicocele-untreated patients there was a statistically significant lower T:E ratio than in the varicocelectomised and control groups (*P* < 0.0001). This ratio significantly improved in the varicocelectomy group during the follow-up (44). Some studies analyzed the hormonal status in patients with varicocele during dynamic testing. In particular, Hudson et al. found a significant difference in free sexual steroid hormones (testosterone, estradiol) and Sex Hormone Binding Globulin (SHBG) pre- and post-varicocelectomy but only in men with excessive responses to Gonadotropins Releasing Hormon (GnRH). In men with higher rise in FSH and LH after GnRH infusion, free testosterone levels were lower, and free estradiol and SHBG levels were higher. After varicocele correction there was a *restitutio ad integrum* (45).

Another study by Castro-Magana et al. showed baseline levels of testosterone, estradiol, and androstenedione (A) in the normal range but different response to hCG stimulation before and after surgery. In particular, testosterone peak after hCG administration was higher after than before varicocelectomy; while estradiol and androstenedione peaks, which were higher before, significantly decreased after surgery (46).

## Discussion

The unfavorable impact of varicocele on semen production has been the primary endpoint of many studies and it has long been recognized. A meta-analysis of Agarwal et al. showed that varicocele was the primary reason for alteration of spermiogram in adult infertile men (47). A more recent trial about varicocele and fertility concluded that varicocele has a growing impact on spermatogenesis: the longer the time since its onset, the greater its effects (48).

On the contrary, the relationship between clinically detectable varicocele and hormonal pattern alterations is still unclear. In some studies hormone alterations are mentioned but the information about their role in clinical practice remains scarce.

From literature, the most investigated hormonal impairment was that of testosterone. We know that varicocele can cause Leydig cells dysfunction in various ways, and it becomes gradually more impaired with the duration of the varicocele. As the result of elevated scrotal temperature, ROS production and hypoxia, inhibition of enzymes involved in sexual steroid biosynthesis lead to decreased intratesticular testosterone production and conversion (49). In this regard, many studies concluded that varicocelectomy significantly improves Leydig cells activity and testosterone production as a result of reversible pathological mechanism.

For example, a trial by Tanrikut et al. showed a statistically significant reduction of testosterone levels in patients with varicocele respect to healthy controls persisting even when analyzed by age, but significantly recovering after surgical correction (32). Although numerous studies have shown testosterone reduction in infertile men with varicocele, there are also studies suggesting that it does not cause any changes. In some cases the adrenal androgen secretion might compensate in the first weeks for the impaired Leydig cells function due to varicocele (50). Moreover, findings in experimental models suggest that short duration varicocele affects intratesticular testosterone firstly and subsequently plasmatic concentrations (50). Since testosterone levels might be impaired, it could be necessary to perform a hormonal test at the first diagnosis of varicocele; in this way, the presence and extent of deficit can be ascertained and corrected if necessary. Little is known about LH and FSH, pituitary hormones which regulate testicular function. Varicocele and subsequently testicular warming can cause decrease of testosterone synthesis by Leydig cells and impairment of germinal and Sertoli cells. A meta-analysis by Tian et al. showed higher FSH and LH levels in varicocele-bearing patients that significantly lowered after surgery (34). Although biochemical screening is suggested as part of the clinical management, data on serum FSH as a surrogate marker for spermatogenesis are conflicting. FSH levels seem to depend on the different steps of spermatogenesis at which the impairment occurs. In fact, they increase only when the azoospermia is related to impaired spermatogonia number and function, whereas they persist in the normal range when the azoospermia is related to maturation arrest at spermatocyte or spermatide stage (8, 51). Evaluation of FSH and LH levels may be necessary at the first diagnosis of varicocele especially if the patient is seeking medical attention for infertility. In fact, although in published literature the correlation between varicocele and infertility is no longer so close, gonadotropins impairment could at least in part explain the alteration of spermatogenesis. About the role of other hormones in patients with varicocele, a significantly lower concentrations of AMH was found in subfertile men, as a result of Sertoli cells damage, without differences related to the grades of subfertility. On the contrary, a progressive serum Inh-B levels reduction was observed from less severe to more severe testicular damage (39). These hormones may be useful indicators in case of increased FSH levels, because they could indicate the existence (AMH, Inh-B) and seriousness (Inh-B) of injure of seminiferous epithelium (39). Decreasing levels of Inh-B could, at least in part, explain the rise in FSH levels in patients with varicocele. The damage of the seminiferous epithelium and germ cells with impaired spermatogenesis causes a decline in Inh-B levels with consequent lack of negative feedback on pituitary FSH secretion. AMH, Inh-B and FSH levels are expression of germinal cells damage; their involvement correlate with more severe testicular damage. Therefore, a recovery of normal values of these hormones after varicocele surgical correction may favor a resumption of normal spermatogenesis which has to be verified by a semen analyses.

Under normal scrotal conditions, a counter-current heat-exchange mechanism between the outflow of the pampiniform plexus and testicular arterial inflow supports the cooler scrotal temperature; in varicocele this physiological mechanism is altered, elevating scrotal temperature. The higher than normal temperature impairs Leydig cells function causing reduced testosterone production. Heat inhibits C-17,20-lyase, the enzyme that converts 17-OH-progesterone to androstenedione and then testosterone, contributing to lowering androgen levels and rising 17-OH-progesterone levels (42). Lower testosterone levels inhibit the (androgen dependent) epididymal 5-α-reductase activity with a consequential reduced conversion to DHT (45). On the other hand the lack of conversion to DHT can causes a rise of aromatization in estradiol. In this regard, some authors suggest the use of aromatase inhibitors for male infertility associated with low testosterone levels and impaired T:E ratio (52), others confirm lower testosterone and T:E ratio in men with varicocele but affirm that these parameters may significantly improve after varicocelectomy (44).

Maintaining normal estrogen concentrations is very important since they play a negative role in the testis by inhibiting Leydig cells 17β-hydroxysteroid dehydrogenase (17β-HSD), and thus inhibiting the conversion of androstenedione to testosterone, lowering testosterone production. Castro-Magana et al. observed an increased androstenedione/testosterone (A/T) ratio after hCG administration before, and its normalization 3 months after varicocelectomy, suggesting an impaired 17β-HSD activity in males with varicocele (46). On the basis of these data, at diagnosis, it could be useful to evaluate also estradiol levels to assess T:E ratio. Its impairment may underlie infertility, which if timely treated, could restore physiological wellbeing and improve the fertility rate.

Finally, as suggested in many studies, SHBG levels in varicocele patients are higher than normal; the cause is unknown, but it may contribute to reduction of free androgen levels.

## Conclusions

Although recent findings have shown a high prevalence of varicocele in men even without impaired fertility, there is no doubt that varicocele could led to hormonal alterations. Different behaviors in affected patients may likely be influenced by individual and/or environmental factors. This review suggests that the impaired free sexual steroid levels are the result of a slight, deep-rooted defect in the testes of a certain amount of men with varicocele, and as usual in endocrinology, the alteration of one hormone leads to a cascade of hormonal alterations owing to inactivating feedback pathways, enzyme inhibition or overactivity and other biochemical mechanisms. The pathological process may be reversible, as demonstrated by surgical correction. Hormonal assessment together with semen analysis should be recommended in all patients with varicocele.

To date, other multicentre, randomized controlled studies remain mandatory to ascertain the hormonal features of patients with varicocele and to assess the utility of hormonal evaluation for establishing the duration of varicocele and for better identifying those patients who need surgical correction.

## Author Contributions

All authors contributed to research data from published papers on this issue for the review, to discuss critically the content, to write the manuscript, and edit the draft before submission. Thus, obviously all of them have read and agree to the final version of the manuscript.

## Funding

Open Access funding provided by Università degli Studi della Campania Luigi Vanvitelli.

## Conflict of Interest

The authors declare that the research was conducted in the absence of any commercial or financial relationships that could be construed as a potential conflict of interest.

## Publisher's Note

All claims expressed in this article are solely those of the authors and do not necessarily represent those of their affiliated organizations, or those of the publisher, the editors and the reviewers. Any product that may be evaluated in this article, or claim that may be made by its manufacturer, is not guaranteed or endorsed by the publisher.
